# Risk factors for intraoperative greater trochanteric fractures in hemiarthroplasty for intracapsular femoral neck fractures

**DOI:** 10.1007/s00068-020-01549-0

**Published:** 2020-12-11

**Authors:** Johannes Karl Maria Fakler, Alexander Brand, Christian Lycke, Christina Pempe, Mohamed Ghanem, Andreas Roth, Georg Osterhoff, Ulrich Josef Albert Spiegl, Andreas Höch, Dirk Zajonz

**Affiliations:** 1 Department of Orthopaedic, Trauma and Plastic Surgery, University Hospital of Leipzig, Liebigstr. 20, 04105 Leipzig, Germany; 2 Department of Orthopaedic and Trauma Surgery, Zeisigwald Hospitals Chemnitz, Chemnitz, Germany; 3 ZESBO-Center for Research on Musculoskeletal Systems, Semmelweisstrasse 14, 04103 Leipzig, Germany

**Keywords:** Hemiarthroplasty, Femoral neck fracture, Greater trochanteric fracture, Periprosthetic fracture

## Abstract

**Purpose:**

Hemiarthroplasty is widely accepted as the treatment of choice in elderly patients with a displaced intracapsular femoral neck fracture. Intraoperative greater trochanteric fractures thwart this successful procedure, resulting in prolonged recovery, inferior outcome, and increased risk of revision surgery. Hence, this study analyzed factors potentially associated with an increased risk for intraoperative greater trochanteric fracture.

**Methods:**

This retrospective study included 512 hemiarthroplasties in 496 patients with a geriatric intracapsular femoral neck fracture from July 2010 to March 2020. All patients received the same implant type of which 90.4% were cemented and 9.6% non-cemented. Intra- and postoperative radiographs and reports were reviewed and particularly screened for greater trochanteric fractures.

**Results:**

Female patients accounted for 74% and mean age of the patients was 82.3 (± 8.7) years. 34 (6.6%) intraoperative greater trochanteric fractures were identified. In relation to patient-specific factors, only a shorter prothrombin time was found to be significantly associated with increased risk of intraoperative greater trochanteric fracture (median 96%, IQR 82–106% vs. median 86.5%, IQR 68.8–101.5%; *p* = 0.046). Other factors associated with greater trochanteric fracture were a shorter preoperative waiting time and changes in perioperative settings. Outcome of patients with greater trochanteric fracture was worse with significantly more surgical site infection requiring revision surgery (17.6% vs. 4.2%, *p* = 0.005).

**Conclusion:**

Prolonged prothrombin time, a shorter preoperative waiting time, and implementing new procedural standards and surgeons may be associated with an increased risk of a greater trochanteric fracture. Addressing these risk factors may reduce early periprosthetic infection which is strongly related to greater trochanteric fractures.

## Introduction

Hemiarthroplasty (HA) is widely accepted as the treatment of choice in elderly patients with a displaced intracapsular femoral neck fracture [1]. It provides good functional results [2, 3] at a fairly low rate of serious complications requiring unplanned secondary procedures in 3–8% [2–4]. Intraoperative periprosthetic fractures in hip arthroplasty are specific complications associated with prolonged recovery, inferior outcome, and increased risk of revision surgery [5–7]. The predestinated location of intraoperative iatrogenic fractures is the greater trochanter with an incidence of approximately 3–5% [8–10]. Although beneficial results after greater trochanteric fractures (GTFs) in individual reports are described [11], persistent pain, a limping gait or significant functional impairment affects 29–65% of these patients [8–10].

In the fragile group of patients with femoral neck fractures sequelae of GTF might even have more pronounced negative effects. With respect to HA, only limited data are available on intraoperative periprosthetic fractures [12–14]. Data analyzing risk factors specifically for intraoperative GTF and their potential effect on outcome are lacking in this group of patients. Subsequently, the primary aim of this study was to identify of risk factors for intraoperative GTF. The secondary aim focused on early outcome parameters in relation to patients without intraoperative GTF.

## Methods

### Patient selection and ethical statement

For this retrospective single-center cohort study, all consecutive patients primarily treated with bipolar HA due to an intracapsular femoral neck fracture were included between July 2010 and March 2020. Patients presenting with a pathologic fracture, prior internal fixation for femoral neck fracture, or presenting with a neglected fracture older than 4 weeks were excluded.

The study was approved by the local Ethics Committee at the University of Leipzig (044/14032016). Written informed consent was obtained from all patients or their legal representative.

### Data collection

Medical charts of all patients were reviewed and demographic data, past medical history, comorbidities, the American Society of Anaesthesiologists (ASA) score, medication, and preoperative routine laboratory blood tests were recorded. From operative reports, description of intraoperative periprosthetic fractures, time and duration of surgery, as well as type of implants were documented.

Median follow-up was 6 (0–23) months and a minimum follow-up of 30 days or until death within 30 days was available in 77.1% of all HAs.

### Surgical treatment

Indications for HA were made in accordance with national guidelines. Surgery was performed in general anesthesia and all patients received a single-shot preoperative antibiotic prophylaxis with cefuroxime or clindamycin in case of history of allergy to penicillin. Patients were placed in a supine position. A modified Hardinge approach was applied in 90.8% and an anterolateral approach in 9.2%. A bipolar hemiprosthesis (Corail stem, bipolar metal head, DePuy Synthes, Warsaw, USA) was the standard implant at our institution. Cemented and non-cemented stems were used in 90.4% and 9.6%, respectively. Because the stem design and corresponding rasp were demonstrated to influence occurrence of intraoperative periprosthetic fractures [13], we excluded 11 HAs that sporadically were performed with other implants than the Corail stem. Regular day time surgical service was provided from 8 a.m. to 8 p.m., on-call service from 8 p.m. to 8 a.m. Postoperatively, patients were mobilized with full weight-bearing provided that they were ambulatory.

49 surgeons performed a hemiarthroplasty in these patients during the observed period of time. Surgeons that performed at least 100 elective hip replacements and continued to be involved in elective hip replacement were defined as hip arthroplasty surgeons (HAS).

Until 2014, hip hemiarthroplasty for intracapsular femoral neck fracture was performed by orthopedic trauma surgeons. In 2014, the formerly separated departments of trauma orthopedic surgery and (elective) orthopedic surgery joined together. Since then, orthopedic surgeons, which primarily were involved in elective hip replacements, were engaged in operative treatment of trauma patients. These surgeons used different hip arthroplasty implants for elective hip replacement before 2014. In 2016, a certified center of arthroplasty was established requiring implementation of additional quality standards with reference to clinical pathways, qualification of surgeons and quality management.

### Radiographic evaluation

Preoperative, intraoperative, as well as postoperative standard anteroposterior pelvis and axial hip radiographs were analyzed by an experienced senior surgeon. All identified greater trochanteric fractures were confirmed by a second experienced senior surgeon. Preoperative and postoperative radiographs were available for review in 100% and 99%, respectively.

### Statistical analysis

Continuous variables were analyzed for normal distribution applying the Kolmogorov–Smirnov test in addition to q–q diagrams. Normally distributed parameters were given as mean and standard deviation (SD); for non-normally distributed parameters, median and the interquartile range [25th–75th percentile] were used. Continuous comparisons were performed using the *t* test for normally distributed variables and the Mann–Whitney *U* test for non-normally distributed variables. For categorical comparisons, according to group size, Chi-squared or Fisher’s test were performed. All statistical computations were performed using SPSS version 24.0 (Chicago, IL, USA). *p* values less than 5% were considered as significant.

## Results

**Fig. 1:**
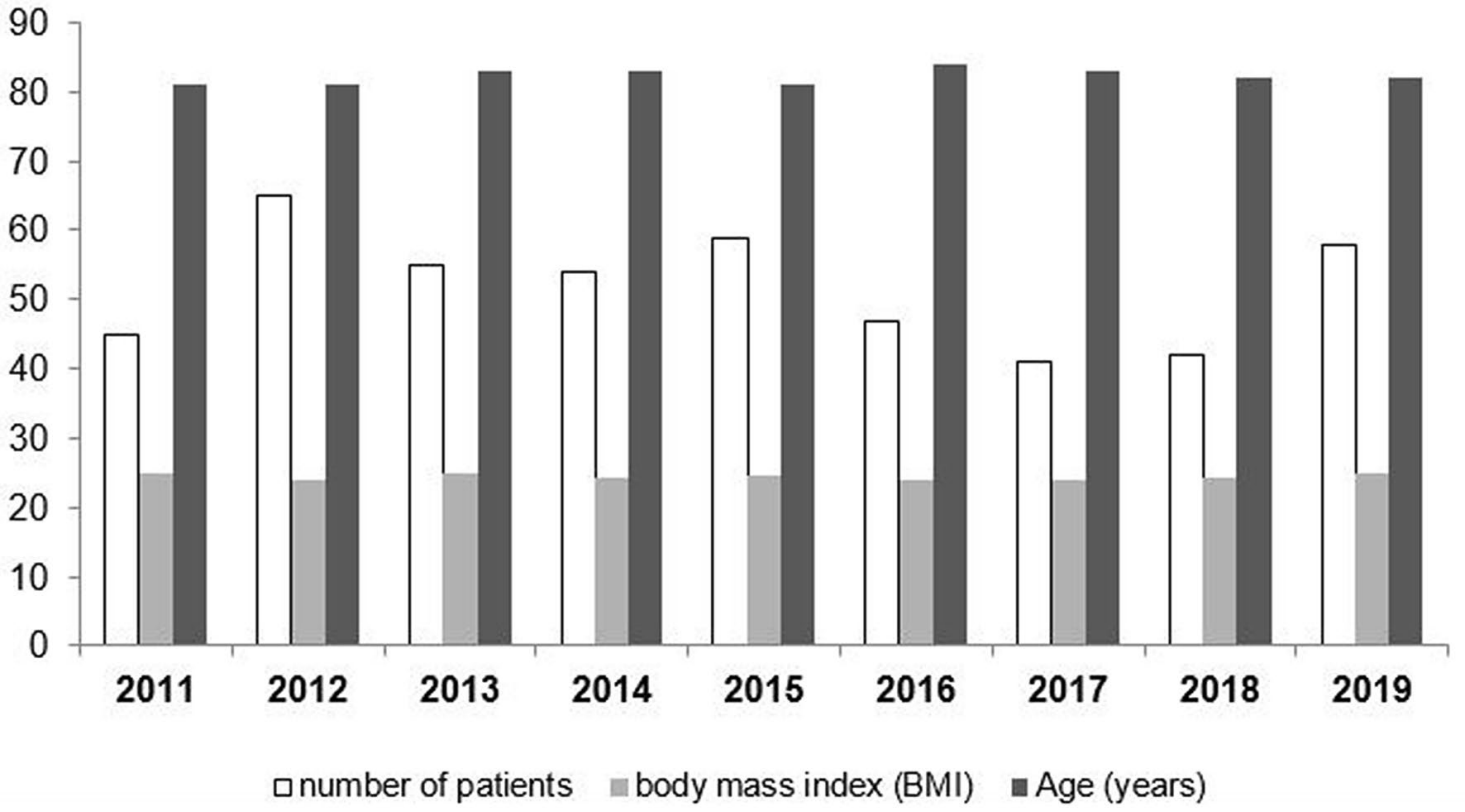
Anual case load and patient characteristics during study period

496 patients with 512 intracapsular femoral neck fractures were treated with HA and met inclusion criteria. Female patients accounted for 74% and mean age of the patients was 82.3 (± 8.7) years. 30-day mortality was 8.8%. During the inclusion period from 2010 to 2020, number of patients, mean patient age, and body mass index (BMI) remained constant (Fig. 1). Surgery was performed on call in 18.9% and within 24 h in 48.4% of all HAs.

Operative reports as well as intraoperative and postoperative radiographs revealed 34 GTFs (6.6%). Only one GTF was fixed intraoperatively by a cerclage wire; no secondary procedures for greater trochanteric fixation were performed.

To identify risk factors, the GTF group was compared to controls without isolated fracture of the greater trochanter. With regard to patient-related factors, only prothrombin time was found to be significantly lower in the GTF group. All other patient-related parameters displayed no differences between groups (Table 1).

**Table 1:** Patient-related factors associated with risk of intraoperative greater trochanteric fracture (GTF)

	Controls (no GTFx, *n* = 478)	GTFx (*n* = 34)	*p*
Age (years)	82.4 ± 8.6	81.1 ± 10.0	0.471
Female sex	357 (75%)	24 (71%)	0.684
BMI	24.4 (22.0–27.0)	23.5 (22.0–26.8)	0.546
Platelet inhibitors	157 (33%)	9 (27%)	0.460
Oral anticoagulants	84 (18%)	7 (21%)	0.817
Warfarin	44 (9%)	5 (15%)	0.358
NOAKs	40 (8%)	2 (6%)	0.759
Diabetes	129 (27%)	7 (21%)	0.472
Smoking	58 (12%)	1 (3%)	0.160
Oral glucocorticoids	29 (6%)	0 (0%)	0.246
CRP (mg/l)	5.6 (1.6–24.7)	7.7 (1.9–28.0)	0.622
Leucocyte count (exp 9/l)	10.2 (8.0–13.2)	11.4 (8.6–15.1)	0.149
25OH D3 (ng/ml)	8.0 (5.0–13.1)	5.2 (4.0–13.1)	0.194
Creatinine (µmol/l)	80.0 (64.0–104.0)	77.5 (64.0–106.5)	0.770
Hemoglobin (mmol/l)	7.7 (6.9–8.4)	7.7 (6.8–8.4)	0.878
Prothrombin time (%)	96 (82–106)	86.5 (68.8–101.5)	0.046
ASA 3 and 4	352 (74%)	27 (79%)	0.547

Values are given as mean (± standard deviation), median (IQR) or absolute numbers and percentage. BMI body mass index, NOAKs non-vitamin K-dependent oral anticoagulants, CRP c-reactive protein, 25OH D3 25-hydroxy vitamin D3

Moreover, risk factors associated with surgery and perioperative settings were analyzed. Preoperative waiting time was significantly shorter in the GTF group (19 h vs 26 h; *p* = 0.006). Most of the HA were performed by attending surgeons in both groups (75% vs. 71%, *p* = 0.683). Although the fraction of hip arthroplasty surgeons (HAS) was lower in the GTF group compared to controls, this difference was not significant (27% vs. 38%, *p* = 0.203). 347 HAs were performed before and 165 HAs after the establishment of a center of arthroplasty at our department with implementation of additional quality standards. Accordingly, only 31% of HAs in the control group with no GTF accounted for the younger period of the hip arthroplasty center. However, number of GTFs distributed equally between these periods with 50% in each (31% vs. 50%, *p* = 0.046) (Table 2). The number of different orthopedic surgeons was 38 before and 29 after implementing the hip arthroplasty center. Nevertheless, the average case load was substantially higher in the earlier period with 9.1 compared to 5.7 HAs per surgeon.

**Table 2:** Surgical and perioperative factors associated with risk of intraoperative greater trochanteric fracture (GTF)

	Controls (no GTFx, *n* = 478)	GTFx (*n* = 34)	*p*
Preoperative time (h)	26 (16–42)	19 (9–30)	0.006*
Duration surgery (min)*	71 (57–84)	75 (61–92)	0.142
Cemented	430 (90%)	33 (97%)	0.235
Surgery on call	91 (19.0%)	6 (17.6%)	1.000
Attending surgeon	359 (75%)	24 (71%)	0.683
Hip arthroplasty surgeon (HAS)	181 (38%)	9 (27%)	0.203
Established center of arthroplasty	148 (31%)	17 (50%)	0.024*

Values are given as median (IQR) or absolute numbers and percentage, * p< 0.05

Patients suffering an intraoperative GTF noticeably affected early postoperative results to the worse (Table 3). Length of hospital stay (LOS) was longer and the rate of postoperative hematoma and seroma requiring further revision surgery tended to be higher (8.8% vs. 2.5%, *p* = 0.070). Finally, early deep infections were substantially higher in the GTF group (17.6% vs. 4.2%, *p* = 0.005).

**Table 3:** Postoperative parameters and early complications

	Controls (no GTFx, *n* = 478)	GTFx (*n* = 34)	*p*
Length of stay (LOS)	10.0 (8.0–14.0)	12.5 (9.9–16.5)	0.009*
30-day mortality	43 (9.0%)	2 (5.9%)	0.757
Revision surgery due to hematoma/seroma	12 (2.5%)	3 (8.8%)	0.070
Revision surgery due to early deep infection	20 (4.2%)	6 (17.6%)	0.005*

Values are given as median (IQR) or absolute numbers and percentage, * p< 0.05

## Discussion

Our retrospective study found 34 (6.6%) isolated GTFs which is in line with other studies that found GTFs in 4.1–6.3% following HA in patients with a femoral neck fracture [12–14]. The only patient-related risk factor for intraoperative GTF identified in our study was a decreased prothrombin time. Although more patients were on warfarin in the GTF group, the difference was not significant (15% vs. 9%, *p* = 0.358). However, prothrombin time may have also been influenced by vitamin K deficiency related to malnutrition or medical disease. Vitamin K plays an important role in bone health and is highly prevalent in patients with hip fracture [15, 16]. In addition, 25OH-vitamin D3 levels tended to be lower in the GTF group supporting the hypothesis that reduced bone health may result in increased susceptibility to iatrogenic fractures. Otherwise, a lower prothrombin time may indicate increased bleeding intraoperatively, especially during rasping the intramedullary canal, which may limit a clear view and urge the surgeon to conclude surgery promptly. This in turn increases the stress level of the surgeon, potentially altering performance with a higher rate of GTFs.

In contrast to our study, no differences of 25OH-vitamin D3 levels were found between patients suffering an intraoperative periprosthetic fracture compared to patients without fracture in a study of 271 treated with HA for femoral neck fracture [12]. However, patients in this study presented with mean 25OH-vitamin D3 levels of 20 ng/ml, whereas hip fracture patients in our region exhibit significantly lower levels with a median of 8.4 ng/ml as shown previously [17]. Hong et al. found no difference of T-Scores between groups, but identified Dorr C type femoral canals [18] as a risk factor for intraoperative fractures as did Bellova et al. [14]. Since we focused on isolated GTF, the Dorr types of the femoral canals were not determined. Bellova et al. [14] found female sex as a risk factor for intraoperative iatrogenic fractures in their series of 481 HA. We and Hong et al. [12] could not confirm sex as a risk factor.

Preoperative waiting time was significantly shorter in the GTF group. Shorter preoperative waiting times may limit optimal preparation and assembly of a well-rehearsed team. Although not significantly, HAS were less frequently involved in the GTF group compared to controls in our study. Moreover, 51.6% of all patients were operated beyond 24 h after admission and 81.1% during routine daytime hours at our institution—which more likely allows allocation of a suitable specialized team. However, surgery performed on-call had no influence on GTF as confirmed by others [14].

Although not by significance, highly specialized hip arthroplasty surgeons (HAS) were represented less frequently in the GTF group than in the control group, implicating that surgical experience may be associated with favorable outcome with regard to this specific complication. Nevertheless, no reduction of GTF was observed after establishment of a certified arthroplasty center, despite implementation of additional quality standards with reference to clinical pathways, qualification of surgeons, and quality management. 50% of the GTFs were recorded after implementation of our arthroplasty center, whereas in the non-fracture group, only 31% of HAs were performed in the period of the arthroplasty center. An explanation could be that by restructuring the arthroplasty service new surgeons had to be integrated into acute fracture arthroplasty. They had to adapt to hip implants and instruments specifically used for acute HAs in femoral neck fracture patients, but not for elective total hip arthroplasty at our institution. In addition, a significantly lower number of HAs was implanted during the considerably shorter period of the hip arthroplasty center underlined by a substantially lower case load per surgeon. Moreover, an implant or instrumentation (i.e., rasp) immanent issue might be responsible for the relatively high rate of GTF in this period. This is supported by Laflamme et al. [13] who found an abnormal number of iatrogenic intraoperative fractures following HA for femoral neck fractures after they switched to the same implant inserted at our institution. They observed 22 (6.3%) iatrogenic GTFs in 348 HAs which is almost identical to our results. Other factors potentially affecting the risk of GTF, as, for example, femoral offset influenced by implant size and design, were not analyzed in our study. However, Laflamme et al. [13] found no relationship between implant sizes and GTF. Moreover, only limited information was found in our operation reports regarding the stage of the surgery at what a GTF occurred. On the other side, it is not always possible to differentiate exactly, whether a GTF originated during hip adduction to expose the proximal femoral canal, preparation of the femoral canal, stem insertion, or reduction of the hip after implantation.

Patients with GTFs after THA were demonstrated to experience a worse outcome with limping, increased pain, and functional impairment [8–10]. In addition, Homma et al. [19] hypothesized that even minor GTFs with a small fragment (chip fracture) may result in increased bleeding and formation of hematoma. This is confirmed in our study. Seroma and hematoma requiring surgical revision clearly tended to be more frequently after GTFs. A significant association was shown between GTFs and early postoperative periprosthetic infections requiring revision.

This study is limited by its retrospective design including patients over a long period of time. Thus, it may be subject to changes and confounders not identified. However, annual case load and patient characteristics remained constant (Fig. 1). Another limitation may be that beyond postoperative radiographs usually no additional or routine follow-up radiographs were ordered and thus not considered. Thus, non-displaced GTFs not identified on postoperative radiographs, but dislocating secondarily may have been missed. Further limitations may be seen in the unequal distribution of the two different approaches used as well as not addressing anatomical aspects of the proximal femur (i.e., varus or valgus hip) which may also have an impact on risk of GTF. Finally, the number of patients with a GTF is relatively small compared to the non-fracture group potentially impairing statistical analysis. The strength of the study lies in a high number of patients that received the same type of implant.

## Conclusion

Prolonged prothrombin time, a shorter preoperative waiting time, and implementing new procedural standards and surgeons may be associated with an increased risk of a GTF. Addressing these risk factors may improve outcome, especially by reducing early periprosthetic infection which is strongly related to GTFs.
